# Impacts of a gross anatomy laboratory course on medical students’ emotional reactions in Taiwan: the role of high-level emotions

**DOI:** 10.1186/s12909-021-02923-1

**Published:** 2021-09-11

**Authors:** Ruei-Jen Chiou, Po-Fang Tsai, Der-Yan Han

**Affiliations:** 1 Department of Anatomy and Cell Biology, School of Medicine, College of Medicine, Taipei Medical University, Taipei, Taiwan; 2 Graduate Institute of Humanities in Medicine, College of Humanities and Social Sciences, Taipei Medical University, Taipei, Taiwan; 3 Section of Liberal Arts, Center for General Education, Taipei Medical University, 250 Wu-Xing St., Xinyi District, 11031 Taipei, Taiwan

**Keywords:** Death anxiety, High-level emotion, Ceremony, Silent mentor, Gross anatomy education

## Abstract

**Background:**

Gross anatomy laboratory course at medical school is usually an important learning subject for medical students; however, seeing a cadaver often makes them feel uncomfortable. According to the broaden-and-build theory, positive emotions broaden our inventory of thoughts and actions, and build physical, mental, and social resources. Research on positive psychology found that through direct thanks and positive reframing, people who feel gratitude show fewer depressive symptoms. The present study tried to reduce students’ negative emotions towards cadavers by sequential activities, such as family interviews and an initiation ceremony, which induced gratitude and other positive emotions.

**Methods:**

The Emotional Reactions Towards Cadavers Scale (ERTCS) was used to evaluate medical students’ emotional reactions after they see a cadaver. Third year medical students (*n* = 105) at Taipei Medical University in northern Taiwan completed ERTCS on three occasions within a single semester during academic year 2016. Repeated-measures ANOVA and hierarchical regression analyses were then conducted to identify any changes in the emotional reactions of these students.

**Results:**

The ERTCS showed satisfactory internal consistency and a three-factor structure, i.e., negative emotions, high-level emotions, and excited emotions. High-level emotions were the highest, and negative emotions were the lowest among the three in our sample. Three-wave data showed that participants’ high-level emotions increased, negative emotions decreased, and the former simultaneously predicted the latter after controlling for the influence of gender, religious beliefs, experience of the death of a family member or friend, and burnout level.

**Conclusions:**

While past research usually focused on coping strategies to reduce medical students’ negative emotions, our study supported the broaden-and-build theory, which emphasizes positive emotions, and demonstrated that elevating medical students’ gratitude to ‘silent mentors’ is an effective way. It is suggested that combining dissection courses with medical humanities can help students successfully handle negative emotions during a gross anatomy laboratory course.

The gross anatomy laboratory course is an important learning subject for medical students, and how teachers prepare them for the emotional impacts before and during the class is a critical concern for medical education. Most young medical students have no experience with death of a close relative or a friend, so that when they go into a dissection room and see a cadaver, they usually manifest multiple physiological and psychological symptoms. Previous studies showed that medical students in a dissection room experience many physiological [1–7] and psychological problems [1, 2, 5, 8]. Symptoms experienced by more than 20 % of participants are listed in Table 1. Generally, about 30 % of medical students’ experience at least one of these adverse impacts when they take a gross anatomy laboratory course [4].

**Table 1:** Literature review of physiological and psychological problems that students report in the dissection room

Symptom	Author (year)	Sample size (*n*)	Incidence (%)
An impulse to leave the laboratory	Bob et al. (2014) [2]	121	27.2
Daytime flashbacks	Sándor et al. (2015) [8]	733	23.7
Dizziness	Getachew (2014) [1]	206	24
	Bob et al. (2014) [2]	121	42.8
Fainting	Bob et al. (2014) [2]	121	28.2
Feeling sick	Bernhardt et al. (2012) [3]	94	33
Heart palpitations	Bataineh et al. (2006) [5]	145	30.3
Insomnia	Bob et al. (2014) [2]	121	20.7
Irritation of the eyes and throat	Saylam and Coskunol (2005) [6]	242	70.9
	Houwink et al. (2004) [7]	126	21.4
Loss of appetite	Getachew (2014) [1]	206	39
	Bob et al. (2014) [2]	121	26.4
Nausea	Getachew (2014) [1]	206	30
	Bob et al. (2014) [2]	121	53.7
	Dempster (2006) [4]	141	20.8
Often think about dissection	Sándor et al. (2015) [8]	733	33.4
Recall of a cadaver’s image	Bob et al. (2014) [2]	121	57
	Bataineh et al. (2006) [5]	145	30.9
Sweating	Getachew (2014) [1]	206	21
	Bob et al. (2014) [2]	121	23.1
Trembling	Bob et al. (2014) [2]	121	33.9

Researchers have tried to explore the reasons causing medical students to suffer so much. Bernhardt et al. used self-composed questionnaires, including some items about previous experiences with death and dying, to investigate psychological stress of 155 first-year medical students (112 females, 43 males) in Germany [3]. They found that students who had no experience with the death of a relative or friend had higher fears when facing body donors. Another study used a checklist and questionnaires to investigate the sensations or reactions of 425 students (79.6 % females, 20.4 % males) to dissection, and pointed out that students who were not prepared or did not have deep thoughts about life and death during dissection had higher death anxiety [9]. Those two studies suggest that helping students face life and death issues could be a good strategy to reduce students’ negative emotions when they take a gross anatomy laboratory course.

## Teachers’ intervention***s and students’ coping strategies***

There have been many attempts proposed by teachers to lower psychological impacts on students of seeing a cadaver. In one attempt, teachers showed students a video about anatomical information before the beginning of the course to lower students’ anxiety level [10–12]. Another intervention provided senior students’ help with first-year students, and found that fewer of them showed anxiety, light-headedness, headaches, and vomiting, and there were also fewer reactions of discomfort to the smell of cadavers and the dissection room [7]. Another study that guided students in groups to share their emotions and thoughts, found that most of the participating students showed that they respected the body donors because donors were living persons before, or they treated donors as if they were their future patients [13].

Students also develop their own coping strategies including rationalization, focusing on tasks, talking to and staying with peers, religious praying, inspiring themselves with the vision of being medical doctors in the future, or taking a break during dissection [1, 2, 5, 8, 13–15]. Another interesting strategy is that students may name the body donors they are dissecting. Compared to those who did not name the body donors, students who had named the body donors remembered the body donors’ features better, thought of the body donors’ lives when they were alive, and were more welcoming to relatives’ body donation decisions. Also, more than half of participating students wanted to know the life stories of the dissection body donors [16]. All such strategies helped students overcome the unpleasantness of cadaver dissection. It seems that inspiring students’ humanistic spirit can reduce the impacts of seeing a cadaver.

Emotions in the Gross Anatomy Laboratory Course

When students see a cadaver in a gross anatomy laboratory course, they often feel strong negative emotions, but also other positive emotions [4–7, 9, 13–15, 17–21]. Negative and positive emotions manifested by more than 20 % of students are listed in Table 2 [4, 9–11, 17, 18, 20]. However, one study observed that students’ high-level emotions, like respect, gratefulness, and cherishing, were aroused, and negative emotions were suppressed compared to those of Western students [22]. In Eastern cultures, we believe that introducing students to know the donors and their families can increase students’ gratitude, and decrease their negative emotions. Therefore, it is worth understanding the mechanism of medical students’ emotional changes in a gross anatomy laboratory course in an Eastern culture.

**Table 2:** Literature review of negative and positive emotions that students experience in a dissection room

Emotion	Author (year)	Sample size (*n*)	Incidence (%)
**Negative**			
Anxiety	Quince et al. (2011) [17]	156	20.3
	Dempster (2006) [4]	141	33.5
	Saylam and Coskunol (2005) [6]	242	29.7
	Lamdin et al. (2012) [14]	21	NA
	Bati et al. (2013) [21]	486	medium level
	Houwink et al. (2004) [7]	126	26
Apprehension	Quince et al. (2011) [17]	156	37.3
Confusion	Quince et al. (2011) [17]	156	22.9
Fear	Bataineh et al. (2006) [5]	145	30.3
	Kotzé and Mole (2013) [13]	240	20
	Tseng and Lin (2016) [15]	12	NA
Uncertainty	Quince et al. (2011) [17]	156	47.7
Being upset	Tseng and Lin (2016) [15]	12	NA
**Positive**			
Calmness	Arráez-Aybar et al. (2008) [9]	425	33.6
Curiosity	Arráez-Aybar et al. (2008) [9]	425	64.6
	Snelling et al. (2003) [20]	364	4.03 ± 0.06/5
Enthusiasm	Quince et al. (2011) [17]	156	79.7
Being excited	Quince et al. (2011) [17]	156	76.5
	Qamar and Osama (2014) [19]	60	34
	Snelling et al. (2003) [20]	364	3.56 ± 0.07/5
Fascination	Quince et al. (2011) [17]	156	83.7
Interest	Quince et al. (2011) [17]	156	99.3
	Cahill and Ettarh (2009) [18]	166	48.4
	Arráez-Aybar et al. (2008) [9]	425	71.7
	Saylam and Coskunol (2005) [6]	242	36.7
	Qamar and Osama (2014) [19]	60	58
	Snelling et al. (2003) [20]	364	4.26 ± 0.06/5
Satisfaction	Arráez-Aybar et al. (2008) [9]	425	25.5

## The broaden-and-build theory

The broaden-and-build theory in positive psychology can explain the mechanism of emotional changes. According to this theory, positive emotions broaden thought-action mechanisms, whereas negative emotions narrow them [23]. Positive emotions can cause individuals to expand their personal cognition and encourage them to have novel, varied, exploratory thoughts and actions. To elaborate, positive emotions broaden one’s point of view, visions, mindsets, experiences, and knowledge of the world, and build resources like skills, physical strength, mental resilience, and social relationships. For example, chasing play of a juvenile animal not only broadens its awareness of the environment, but also builds skills for future escape from predators and hunting prey. Children’s play broadens the ability to control muscles, and builds social relationship and intellectual development. These actions generate positive adaptive circulation and have lasting benefits. On the contrary, negative emotions are viewed as an adaptation when an organism faces danger; they narrow thought-actions to let an organism focus on attacking or escaping for survival. Thus, positive emotions create much more positive effects in the learning process [23–25].

Gratitude, i.e., feelings of thankfulness and appreciation when someone receives another’s kindness and help, has the same broaden-and-build features as other virtues such as wisdom, knowledge, courage, humanity, justice, and temperance studied in the field of positive psychology. These feelings broaden people’s action to promote the well-being of others without limitations to the person who benefited them. They also build people’s social connections and friendships [23]. Previous research pointed out that positive emotions undo the effects of negative emotions [26]. Research on positive psychology found that through direct thanks and positive reframing, people who feel gratitude showed fewer depressive symptoms [27]. Another study with an experimental and control group design showed that inducing gratitude can also decrease death anxiety [28].

## ***Viewpoints of c***onstructivism ***in education***

Constructivism in education is a student-centered learning approach in which the individual creates or builds his or her own understanding or knowledge by interacting with the environment [29]. In the processes of teaching and learning, students take responsibility for their own learning, and teachers are just mediators. We believe that through contacting with family members and/or friends of body donors, students can expand their thoughts and actions, by not only understanding why body donors would sacrifice themselves to help medical students in learning dissection, but also inspiring their motivation to help patients in the future. In addition, medical students can build their own resources by learning communication skills and expressing thankful feelings. In the ceremony for the body donors, students further promise to study hard and become good doctors. Expanding students’ gratitude during the time they take the gross anatomy laboratory course not only inspires other positive emotions like respect, cherishing, and humility, but also helps them spontaneously transcend the original negative emotions like fear, being spooked, and escape. From the viewpoint of constructivism, experiencing the entire process is more effective than listening to a teacher on the podium a hundred times.

Gratitude can be conceptualized on two levels: state and trait. State gratitude is an immediate effect after an event, while trait gratitude is more like a personal characteristic [30]. State gratitude might have a meaningful effect on decreasing medical students’ death anxiety and related negative emotions by inducing gratitude toward the body donors. Thus, we designed family interviews and an initiation ceremony to examine the effects on students’ emotions.

## Factors of the adverse effects

In terms of potential factors of the aforementioned impacts, research has shown that female students reported more threatening feelings [4] and greater reluctance to cut open a cadaver [17]. When students were asked to express their feelings about dissection, male students reported more anxiety and excited feelings, whereas females reported more uncertainty and worry [17]. It was also reported that female students showed more mental distress than males did before and after they interacted with a cadaver [31]. Those studies suggested that female students have more negative physiological and psychological impacts than do male students, and take more time to recover. In terms of religious influence, a qualitative study showed that students with religious beliefs were able to endure the impacts of dissection with help from their beliefs [14]. Other research pointed out that some students pray or meditate as a coping strategy [1, 13]. It is plausible that religious beliefs can help medical students get through adverse effects of a gross anatomy laboratory course. In addition, when students feel exhausted, their emotions become more negative [32, 33]. Having experienced the death of a friend or relative is also a factor that influences students’ reactions to cadavers [3]. Therefore, factors such as gender, religious beliefs, experience of the death of others, and one’s personal exhaustion level should be controlled for to promote the accuracy when carrying out such a study.

### Study goals

Based on the above inferences, the aims of the present study were to determine whether activities such as family interviews and an initiation ceremony that expand medical students’ gratitude toward ‘silent mentors’ increase their high-level emotions and decrease their negative emotions, and whether high-level emotions predict negative emotions after controlling for variables including gender, religion, experience of the death of a family member or friend, and burnout level. To further clarify our goals, the research questions and our hypotheses are listed in Table 3. Although we matched research questions with research hypotheses, it is crucial to claim a post-positivism standpoint as the epistemological stance of this study [34, 35]. Our research design included different approaches, combining an experimental intervention with quantitative data collection. In addition, our quantitative analyses contained three different statistical techniques which were respectively deployed according to the results we sequentially attempted to establish. A post-positivist epistemology not only determined our study design, methods, and data analyses, but also influenced the way we developed our discussion, limitations, and conclusions.

**Table 3:** Research questions and hypotheses of the present study

No.	Research question	Hypothesis
1.	Do activities such as family interviews and an initiation ceremony that emphasizes gratitude actually increase students’ high-level emotions?	Activities guiding students to have thankful thinking towards the ‘silent mentors’ can increase students’ high-level emotions.
2.	Do high-level emotions function to decrease students’ negative emotions?	High-level emotions can decrease students’ negative emotions.

## Materials and methods

### Participants

Participants consisted of 60 male and 45 female third year medical students (*n* = 105) at Taipei Medical University in northern Taiwan. These students chose the science domain in senior high school, and entered medical university for a 6-year Doctor of Medicine (MD) degree under high expectations from their parents and teachers, and high competition with cohorts. In the gross anatomy laboratory course, every 20 students gets a single cadaver to practice dissection in one semester. Owing to the limited number of cadavers each year, these students treasured the opportunity to operate with their own hands, and generally were grateful to cadavers and their families [22]. They also tried their best to learn and hoped that they would pass qualifying exams to become a physician, who possesses high social status in Taiwan. The age of participants ranged 20 ~ 27 (mean ± SD: 20.81 ± 1.10) years. In total, 46 participants had religious beliefs, and 68 of them had had an experience of the death of a friend or family member.

We collected data in their third year when they took the gross anatomy laboratory course; however, these students had gradually received teaching about gratitude toward ‘silent mentors’ since the start of their year two curriculum in medical school. Their lecturers had instructed them to think of the contribution of ‘silent mentors’ and the knowledge that they would gain from the ‘silent mentors’. During the gross anatomy laboratory course, students spent 8 hours a week in a dissection room for 18 weeks. They spent an extra 8 hours reviewing body structures in the dissection room before the midterm and final exams.

### Our Intervention

We instituted a series of activities to accompany our gross anatomy laboratory course at Taipei Medical University since 2010. Teachers and students conduct face-to-face interviews with the family members and/or friends of body donors at their houses or in a meeting room at the university. Students can then practically understand that the body donor on which they will work is not just a teaching instrument in a laboratory but was previously a living person. In the interview, students asked many questions about what kind of person the donor was, what he or she liked or hated, and why she or he decided to be a body donor. Through the interview, students could also understand that the family members and/or friends of the body donor would expect them to become excellent medical practitioners. After interviews of all groups, the Department of Anatomy and Cell Biology organizes an initiation ceremony in September at the beginning of the gross anatomy laboratory course with medical students, faculty members, and some relatives of the body donors in an auditorium at the university. The ceremony is carried out according to general Taiwanese rituals, without religious features, and cadavers are not exposed on-site. Students show their appreciation to the relatives, and promise that they will do their best to be good medical doctors in the future. In Taiwan, students and faculty of medical schools often call a body donor a ‘silent mentor’, who teaches students through donating his or her body. Therefore, students are learning with a teacher who does not speak. We expected that our intervention might substitute students’ negative emotions or psychological impacts with respect for the ‘silent mentors’. On the other hand, based on feedback from our past interviews with students, an initiation ceremony can possibly fortify students’ serious attitudes towards the gross anatomy laboratory course, as well as foster a thankful attitude to the body donors and their relatives.

### Measures

#### Emotional Reactions Towards Cadavers Scale (ERTCS)

Based on students’ descriptions after the first time they saw a cadaver and their emotional reactions observed by leaders of the Gross Anatomy Laboratory course, our research team listed 25 main emotions to measure students’ emotional reactions after they see a cadaver. Items of the ERTCS are rated from 1 (strongly disagree) to 7 (strongly agree) on a Likert scale [22].

In order to categorize major emotional changes of students and validate the ERCTS, we conducted a pilot study that consisted of other 96 male and 62 female medical students (*n* = 158) at Taipei Medical University in 2015. Their ages ranged 19 ~ 30 (20.97 ± 1.53) years. The validity and reliability of the ERCTS are given in the ‘Results’ section.

#### Self-Perceived Physical and Mental Condition and Relationship with their Families

We asked participants to report their self-perceived physical and mental conditions, ranging from 1 (very good) to 5 (very tired). We also asked participants to report their relationships with their families, ranging from 1 (very good) to 5 (very bad).

#### Experience of the Death of a Family Member or Friend and Demographic Variables

We employed a yes/no question to ask participants whether they had had any experience of the death of a family member or friend. In addition, we collected participants’ basic demographic variables including age, gender, and religious beliefs.

### Procedures

#### Research Procedures

**Fig. 1:**
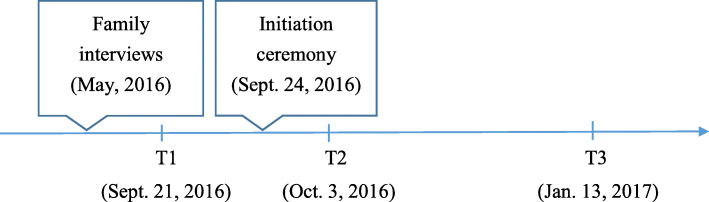
Timeline of family interviews, initiation ceremony, and three-wave data collection.

In their third year in medical school, participants filled out a measure of emotions and self-perceived physical and mental conditions three days before the ‘silent mentor’ initiation ceremony [time 1 (T1), on September 21, 2016]. They again filled out the emotional measurement form after the ceremony (T2, on October 3, 2016). To evaluate changes at the end of the semester, they also filled out the emotional measurement form at the end of the semester (T3, on January 13, 2017). A timeline of family interviews, initiation ceremony, and three-wave data collection is shown in Fig. 1.

#### Ceremony Procedures

Participants in the initiation ceremony included family members and friends of the ‘silent mentors’, all of the third-year medical students, and faculty members of the Department of Anatomy and Cell Biology. In the ceremony, each group of students was told about the silent mentors’ past conduct and deeds to commemorate and appreciate them, and a wreath was laid.

#### Data Processing

We first conducted descriptive statistics including mean and standard deviation (SD) to show the distribution of the three emotions at different time points. Then we performed a repeated-measures analysis of variance (ANOVA) to examine whether the levels of the three emotions changed with time [36]. Finally, two hierarchical regression analyses were used to examine whether participants’ high-level emotions at T2 and T3 could respectively predict their negative emotions at T2 and T3 when the participants’ gender, religious beliefs, experience of the death of another, high-level emotions, and negative emotions at T1 were controlled for.

#### Ethics

The present study received ethical approval from the TMU-Joint Institutional Review Board (no. N201602066), and all methods were performed in accordance with relevant guidelines and regulations. Students volunteered to participate in the study, and informed consent was obtained from all participants. To avoid students’ concerns about filling out the questionnaires, the instructions of the questionnaires stated that the results would not influence their scores, and they only needed to provide their student ID rather than their names to decrease their uncertainty.

## Results

Prior to illustrating the main findings, the structure and consistency of the ERTCS are shown using data from our previous study [22]. To validate the ERTCS, we conducted an exploratory factor analysis, with the method of maximum likelihood factoring. The Kaiser-Meyer-Olkin measure of sampling adequacy (0.86) and Bartlett’s test of sphericity test [*χ*^2^(300) = 2140.96, *p* < 0.001] for the correlation matrix showed that these data were suitable for a factor analysis [37]. The criterion of a eigenvalue > 1 and Cattell’s scree plot [38] were both considered to decide the number of axes, and the rotation method of promax was adopted as these emotions should be theoretically inter-correlated [39]. We found that a three-factor structure was the most meaningful result (Table 4), and 49.47 % of the total variance was accounted for by the three factors, namely negative emotions (e.g., fear, being terrified, and being spooked, 23.40 %), high-level emotions (e.g., respect, reverence, and gratitude, 20.14 %), and excited emotions (e.g., full expectations, happiness, and curiosity, 5.93 %). As for reliability, all of the 25 items showed high consistency (Cronbach’s α = 0.85) [40]. Deleting any single item did not considerably elevate the consistency. Cronbach’s α values of the three factors were 0.88, 0.90, and 0.84, respectively. The ERCTS showed satisfactory validity and reliability. Thus, we further explored changes in these three emotions at the three time points.

**Table 4:** Factor structures of the Emotional Reactions Towards Cadaver Scale

	Factor		
	Negative emotions	High-level emotions	Excited emotions
Fear	**0.79**	-0.11	-0.08
Being terrified	**0.76**	-0.14	0.09
Being spooked	**0.74**	-0.23	-0.01
Shock	**0.68**	0.03	0.07
Pain	**0.67**	-0.18	-0.02
Feeling low	**0.63**	-0.21	-0.14
Being unlucky	**0.59**	-0.37	-0.04
Avoidance as a taboo	**0.59**	-0.19	0.04
Feeling like escaping	**0.59**	-0.24	-0.34
Grief	**0.50**	0.20	0.18
Nervousness	**0.46**	0.27	0.26
Too sympathetic to bear	**0.39**	0.26	0.24
Doubt (to future processes on a cadaver)	**0.35**	0.15	0.32
Disappointment (an inability to achieve others’ expectations)	**0.35**	0.06	0.07
Respect	-0.12	**0.89**	0.43
Cherishing	-0.08	**0.88**	0.50
Gratitude	-0.18	**0.85**	0.57
Admiration	-0.10	**0.82**	0.48
Humility	-0.04	**0.77**	0.34
Peace	-0.40	**0.48**	0.36
Full expectation	-0.03	0.51	**0.80**
Happiness	-0.01	0.51	**0.77**
Curiosity	0.06	0.36	**0.72**
Familiarity (with the ‘silent mentor’)	-0.02	0.56	**0.67**
Excitement	0.10	0.19	**0.62**
Eigenvalue	5.85	5.04	4.48
% of variance explained	23.40	20.14	5.93
Cronbach’s α-coefficient	0.88	0.90	0.84

**Fig. 2:**
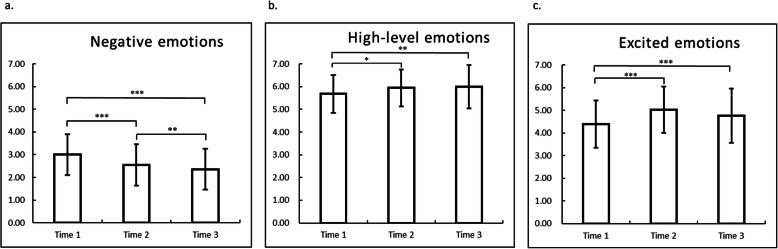
Changes and significance levels of the three emotions (**a**. negative emotions; **b**. high-level emotions; **c**. excited emotions) at three time points. * *p* < 0.05, ** *p* < 0.01, *** *p* < 0.001.

Descriptive statistics are listed in Table 5, and emotional changes and significance levels are shown in Fig. 2. In general, participants reported high values for high-level emotions, moderate values for excited emotions, and low values for negative emotions.

**Table 5:** Descriptive statistics and repeated-measures analysis of variance (ANOVA) of the three emotions at three time points and self-perceived conditions

	Time 1		Time 2		Time 3		Repeated-measures ANOVA		
	Mean	SD	Mean	SD	Mean	SD	*F*	*df*	*p*
Negative emotions	3.00	0.89	2.54	0.91	2.36	0.90	22.62	2,160	< 0.001
High-level emotions	5.68	0.83	5.95	0.81	5.99	0.95	6.05	2,160	< 0.001
Excited emotions	4.39	1.04	5.02	1.02	4.77	1.20	15.96	2,160	< 0.001
Self-perceived condition	1.67	0.71							

Emotions were rated by the Emotional Reactions Towards Cadavers Scale with a Likert-type scale from 1 (strongly disagree) to 7 (strongly agree)

*SD* standard deviation

We conducted a repeated-measures ANOVA to examine whether the three types of emotions changed with time. The analysis observed significant within-subject effects on negative emotions [F (2, 160) = 22.62, *p* < 0.001], and post-hoc comparisons with the Bonferroni correction revealed significant differences among T1 (3.00 ± 0.89) contrasted with T2 (2.54 ± 0.91, *p* < 0.001), T2 contrasted with T3 (2.36 ± 0.90, *p* < 0.01), and T1 contrasted with T3 (*p* < 0.001). In addition, significant within-subject effects on high-level emotions were observed [F(2,160) = 6.05, *p* < 0.01], and post-hoc comparisons with the Bonferroni correction revealed significant differences among T1 (5.68 ± 0.83) contrasted with T2 (5.95 ± 0.81, *p* < 0.05) and T1 contrasted with T3 (5.99 ± 0.95, *p* < 0.01). Also, a significant within-subject effects on excited emotions was found [F(2,103) = 15.96, *p* = 0.001)], and post-hoc comparisons with the Bonferroni correction revealed significant differences among T1 (4.39 ± 1.04) contrasted with T2 (5.02 ± 1.02, *p* < 0.001), and T1 contrasted with T3 (4.77 ± 1.20, *p* < 0.001). Results showed that participants had fewer negative emotions after they participated in the ceremony (T1 vs. T2, *p* < 0.001), and even fewer negative emotions at the end of the semester (T2 vs. T3, *p* < 0.01). Participants had significantly more high-level emotions after they participated in the ceremony (T1 vs. T2, *p* < 0.05), but with no significant change at the end of the semester (T2 vs. T3, *p* > 0.05). Participants had significantly more excited emotions after they participated in the ceremony (T1 vs. T2, *p* < 0.001), and an insignificant drop in their excited emotions at the end of the semester (T2 vs. T3, *p* > 0.05). In short, after the ceremony, participants had fewer negative emotions, more high-level emotions, and more excited emotions. At the end of the semester, their negative emotions continued to drop, high-level emotions remained high, and excited emotions had slightly rebounded.

Last, we conducted two hierarchical regression analyses to examine whether participants’ high-level emotions at T2 and T3 would respectively predict their negative emotions at T2 and T3 when participants’ high-level emotions and negative emotions at T1 were controlled for. Results are shown in Tables 6 and 7.

**Table 6:** Hierarchical regression analyses of high-level emotions to negative emotions at T2

Model 1: Regression of T2 on negative emotions			b		β		*p*
Level 1	Constant	2.719				< 0.001	
Δ*R*^2^ = 0.016 ΔF = 0.398 *p* = 0.810	Have religious beliefs	-0.168		-0.095		0.354	
	Having experienced the death of another	-0.067		-0.036		0.721	
	Self-perceived condition	0.038		0.028		0.785	
	Gender	-0.120		-0.068		0.504	
Level 2	Constant	1.878				0.007	
Δ*R*^2^ = 0.287	Have religious beliefs	-0.214		-0.122		0.167	
ΔF = 19.542	Having experienced the death of another	0.012		0.007		0.940	
*p* < 0.001	Self-perceived condition	-0.136		-0.100		0.267	
	Gender	-0.125		-0.071		0.417	
	Negative emotions (T1)	0.547		0.534		< 0.001	
	High-level emotions (T1)	-0.096		-0.095		0.280	
Level 3	Constant	2.870				< 0.001	
Δ*R*^2^ = 0.054	Have religious beliefs	-0.164		-0.093		0.283	
ΔF = 5.412	Having experienced the death of another	0.011		0.006		0.942	
*p* < 0.001	Self-perceived condition	-0.132		-0.097		0.273	
	Gender	-0.144		-0.082		0.341	
	Negative emotions (T1)	0.519		0.508		0.000	
	High-level emotions (T1)	0.025		0.024		0.806	
	High-level emotions (T2)	-0.271		-0.234		0.022

**Table 7:** Hierarchical regression analyses of high-level emotions to negative emotions at T3

Model 2: Regression of T3 on negative emotions		b	β	*p*
Level 1	Constant	2.556		<0.001
Δ*R*^2^=0.007 ΔF=0.163 *p*=0.957	Have religious beliefs	-0.043	-0.025	0.816
	Having experienced the death of another	-0.051	-0.028	0.793
	Self-perceived condition	-0.068	-0.058	0.595
	Gender	-0.060	-0.035	0.748
Level 2	Constant	1.902		0.008
Δ*R*^2^=0.137	Have religious beliefs	-0.062	-0.037	0.718
ΔF=6.889	Having experienced the death of another	-0.021	-0.012	0.906
*p*=0.002	Self-perceived condition	-0.157	-0.134	0.203
	Gender	-0.040	-0.023	0.821
	Negative emotions (T1)	0.365	0.369	0.001
	High-level emotions (T1)	-0.058	-0.062	0.539
Level 3	Constant	3.487		<0.001
Δ*R*^2^=0.113	Have religious beliefs	0.030	0.018	0.856
ΔF=12.963	Having experienced the death of another	0.013	0.007	0.938
*p*=0.001	Self-perceived condition	-0.269	-0.229	0.026
	Gender	-0.045	-0.026	0.787
	Negative emotions (T1)	0.347	0.351	<0.001
	High-level emotions (T1)	0.098	0.104	0.323
	High-level emotions (T3)	-0.382	-0.390	0.001

In both analyses, at level 1, we controlled for confounding variables, including gender, having religious beliefs, self-perceived physical and mental conditions, and having had experienced the death of a family member or friend. At level 2, we controlled for participants’ negative emotions and high-level emotions at T1. At level 3, we regressed participants’ high-level emotions at T2 and T3 on their negative emotions at T2 and T3, respectively. Results showed that participants’ high-level emotions at both T2 and T3 significantly and negatively predicted their negative emotions at T2 and T3.

## Discussion

The present study deployed three different statistical techniques, and sequentially found three important results. First, we distinguished high-level emotions and negative emotions, which respectively were our explanatory variable and response variable, from excited emotions with an exploratory factor analysis. Second, stable changes at three time points—rises in high-level emotions and declines in negative emotions—achieved significance with the repeated-measures ANOVA. Third, after controlling for impacts of external factors, negative emotions could be predicted not only by their previous value, but also by high-level emotions at the same time point according to the hierarchical regression analyses. The consideration of arranging our results in these three steps did not come from statistical concerns, but relied on a post-positivism epistemological standpoint, which determined our study design, methods, and data analyses. In this regard, we provide our discussion of these results as follows.

Although the literature on coping strategies, whether coming from educational interventions or from students’ adoption during the gross anatomy laboratory course, crucial emotional reactions still remain under-elaborated, and related factors undifferentiated [5, 7, 8, 12, 13, 15]. We elaborated this problem with the ERTCS by distinguishing three different emotions—negative, high-level, and excited emotions—and then established a relationship between high-level emotions and negative emotions. Scarcely mentioned in the previous literature [4–7, 9, 13–15, 17–21], our study showed that high-level emotions were relatively high and negative emotions were relatively low. High-level emotions represent an achievement of our educational intervention, and were the main effect of the initiation ceremony and family interviews, while negative emotions were a crucial and refined indicator of medical students’ emotional reactions toward cadavers during the course. With the help of differentiation, we not only viewed high-level emotions as a predictive variable and negative emotions as a dependent variable by separating them from excited emotions, which could be a confounding factor, but also analytically considered both the diachronic relationship within high-level emotions or negative emotions and the synchronic relationship between the 2 types of emotion. Only when the relationship among different emotional reactions were elaborated could we researchers scrutinize the ‘coping strategies’ designed by educators. In other words, we found that the educational intervention, both the initiation ceremony and the family interviews, could raise students’ high-level emotions and decrease students’ negative emotions.

Based on this primary finding, we first demonstrated the question of how students’ high-level emotions can decrease their negative emotions after controlling for demographic factors. Within the trends of high-level emotions continuing to rise and negative emotions continuing to drop from T1 to T2 and T3, the three-step hierarchical or sequential regression analyses showed three crucial points worth discussing, which might echo both the broaden-and-build theory in positive psychology in general and the state-gratitude as a positive psychological motive in particular.

First, external factors, represented by variables we controlled for, such as gender, religion, experience of a death of another, and self-perceived conditions, had no significant effects on students’ negative emotions at T2 or T3. Although our findings did not exclusively reject previous research about different emotional reactions in terms of gender or religious factors [14, 17, 20, 31], those external factors actually had no influences when the diachronic and synchronic relationships between different emotional reactions were considered. This basic finding enabled us at an early point to shift our focus from external factors to internal factors, indicating a series of psychological mechanisms that needed to be explored. With the help of the broaden-and-build theory, we, however, traced the psychological process neither in medical student’s individual coping strategies [1, 2, 5, 7, 8, 13–15], nor in medical educators’ interventional strategies [10–12], but directly in distinguishing student’s emotional reactions in order to find a solution within [16, 22]. On the one hand, after sorting out high-level emotions, we found that many of them, such as ‘respect, gratitude, admiration, and humility’, could be recognized as state-gratitude after the initiation ceremony and the family interview; on the other hand, these emotions might not only ‘broaden’ medical student’s compassion for donor’s family, but also ‘build’ their own confidence in facing cadavers in the present and preparedness for death in the future.

Second, both models 1 and 2 in the hierarchical regression analysis showed that high-level emotions might have a synchronic impact on negative emotions. Results indicated an elaborate relationship between consistently rising high-level emotions and consistently dropping negative emotions. More important to us was the fact that the impact of high-level emotions on negative emotions became stronger (b=-0.271 in model 1; b=-0.382 in model 2), while the impact of previous negative emotions on negative emotions became weaker (b = 0.591 in model 1; b = 0.347 in model 2). This advanced finding supports our research argument: although there is much research on mitigating negative emotions and coping strategies, medical students’ negative emotions gradually decreased, which might have been a foreseeable consequence of students’ adaptation [9–11, 18, 20]. On the contrary, the often-neglected factor, actively and positively reducing students’ negative emotions, should come from another rising factor which was the result of the educational intervention combined with students’ perceptions. In our research findings, consistently rising high-level emotions played a role as an active, positive factor, and filled the vacuum in research on students’ negative emotions. Interestingly, our findings confirmed previous research and somehow extended their conclusions that naming the body donor helped students overcome their uneasiness of seeing a cadaver [16]. In fact, naming and related activities—remembering the donor’s personality, thinking of the donor’s life, or approaching the donor’s relatives—belonged to events that could raise student’s high-level emotion as gratitude. In addition, this also reconfirmed and echoed our previous research that rituals with a humanistic design decrease negative emotions towards cadavers during the learning process [22].

Third, educational interventions such as the initiation ceremony and family interviews might have two different mechanisms mitigating students’ negative emotions. In a directly influencing mechanism, we found that students’ negative emotions decreased after the initiation ceremony and family interviews. Students definitely adapted to the uncomfortable feelings toward the cadaver dissection; however, our previous study indicated that rituals or activities with a humanistic design, such as the initiation ceremony, could significantly facilitate student’s adaptation and efficiently mitigate their uneasiness [22]. However, there might be another yet undetected way, an indirectly influencing mechanism, of mitigation. In this study, we elaborated that on the one hand, high-level emotions increased after the initiation ceremony and family interviews, and high-level emotions also caused a mitigating impact on negative emotions. Only after we established relationships among the educational intervention, high-level emotions, and negative emotions was the indirectly influencing mechanism obviously revealed. This underlying relationship is not only a kind of moral feelings that cultivated student’s knowledge of life education, but also a psychological interaction between high-level emotions and negative emotions, which was coined with the term of either ‘reframing emotion’ or ‘undoing effect’ in positive psychology [23, 24, 26, 27]. In this regard, our finding that high-level emotions can mitigate negative emotions could be interpreted as a kind of ‘reframing negative emotions with high-level emotions’ or ‘undoing negative emotions with high-level emotions’ through state gratitude as a positive psychological motive.

In summary, the present study demonstrated that (1) educational interventions, such as the initiation ceremony and family interviews, can significantly increase high-level emotions and decrease negative emotions; and (2) high-level emotions can significantly decrease negative emotions under a condition in which external factors are controlled. These two related findings indicate an interesting fact that after differentiating emotions into high-level, excited, and negative emotions, we found an internal way of mitigating medical students’ negative emotions during a gross anatomy laboratory course. Trying to elaborate the internal causality between high-level emotions and negative emotions has become a new issue worth noting, while past research usually focused on coping strategies, whether personal or institutional ones [1, 2, 5, 7, 8, 10–15].

Furthermore, high-level emotions based on the ERTCS (see Table 4) echo the concept of gratitude, which had a buffering effect of decreasing negative emotions. We could extend this confirmative finding in advance with the broaden-and-build theory in positive psychology: when high-level emotions show various similarities to state gratitude, they could act as a positive psychological motive during the cadaver dissection course, through both ‘broaden’ and ‘build’ mechanisms, to mitigate students’ negative emotional reactions. This mitigating interaction of high-level emotions on negative emotions performed as much as the ‘reframing emotion’ or the ‘undoing effect’ on other positive emotions [23, 24, 26, 27].

### Limitations

From a research design viewpoint, we should have utilized experimental and control groups to examine the intervention effects; however, students were willing to participate in these activities because of the value and meaning. Thus, a hierarchical regression was used to control for unnecessary influences to compensate for this study design issue. We believe that medical education research cannot always rely on mainstream approaches deployed in a randomized controlled trial; and yet we can gain valuable insights by combining experimental or educational interventions with quantitative or qualitative data. Randomized controlled trials are not the gold-standard methodological approach to medical education research in the way that such trials are in medical science. In this sense, we suggest triangulation which can ensure both validity and reliability.

From comparisons between Taiwan and other countries, medical students in our research sample reflected a lower scope of negative emotions, which implies that there was seemingly no dramatic mitigation of all kinds of negative emotions as has occurred in the contexts of other research. However, to what extent high-level emotions can mitigate negative emotions under paired research conducted in different countries with their own cultural contexts would be an interesting question to explore. Therefore, we suggest that comparisons between Taiwan and other countries be conducted in the future.

The last limitation is that we did not perform a power calculation to identify the adequate sample size prior to executing the study. Considering the cadaver dissection as a constant course, we suggested establishing longitudinal data enabling researchers to testify the long-term effects of positive psychological motives. This will enrich research into cadaver dissection courses with the current situation where a cross-sectional study is the most commonly seen method.

## Conclusions

Combining dissection courses with medical humanities is helpful for students coping with their negative emotions during a gross anatomy laboratory course. Our research findings show that high-level emotions significantly mitigated negative emotions indicating practical implications in medical education by combining body dissection with medical humanities. As suggested by our study, direct deploy biographical information or life story of a ‘silent mentor’ should be used rather than indirectly developing students’ coping strategies. Therefore, holding curricular activities like family interviews or ceremonies is a good strategy to alleviate students’ negative emotions, and they also bring other positive learning effects according to our past study [22]. We believe that the implications of our study will both facilitate instructors’ practice and improve students’ learning effectiveness in gross anatomy laboratory courses.

## Data Availability

The datasets generated and analyzed during the current study are not publicly available due to protection of the privacy of subjects, but are available from the corresponding author upon reasonable request.

## Abbreviations

ANOVA: Analysis of variance
ERCTS: Emotional Reactions Towards Cadaver Scale
SD: Standard deviation
